# An exploration of collaborative scientific production at MIT through spatial organization and institutional affiliation

**DOI:** 10.1371/journal.pone.0179334

**Published:** 2017-06-22

**Authors:** Matthew Claudel, Emanuele Massaro, Paolo Santi, Fiona Murray, Carlo Ratti

**Affiliations:** 1 Lab for Innovation Science & Policy, Massachusetts Institute of Technology, Cambridge, MA, United States of America; 2 Senseable City Laboratory, Massachusetts Institute of Technology, Cambridge, MA, United States of America; 3 Istituto di Informatica e Telematica del Consiglio Nazionale delle Ricerche, Pisa, Italy; 4 MIT Sloan School of Management, Cambridge, MA, United States of America; US Army Engineer Research and Development Center, UNITED STATES

## Abstract

Academic research is increasingly cross-disciplinary and collaborative, between and within institutions. In this context, what is the role and relevance of an individual’s spatial position on a campus? We examine the collaboration patterns of faculty at the Massachusetts Institute of Technology, through their academic output (papers and patents), and their organizational structures (institutional affiliation and spatial configuration) over a 10-year time span. An initial comparison of output types reveals: 1. diverging trends in the composition of collaborative teams over time (size, faculty versus non-faculty, etc.); and 2. substantively different patterns of cross-building and cross-disciplinary collaboration. We then construct a multi-layered network of authors, and find two significant features of collaboration on campus: 1. a network topology and community structure that reveals spatial versus institutional collaboration bias; and 2. a persistent relationship between proximity and collaboration, well fit with an exponential decay model. This relationship is consistent for both papers and patents, and present also in exclusively cross-disciplinary work. These insights contribute an architectural dimension to the field of scientometrics, and take a first step toward empirical space-planning policy that supports collaboration within institutions.

## Introduction

The practices of academic research are customarily structured around well-defined physical spaces and institutional organizations [1–3], yet the sited collaboration networks within communities are not well understood [4,5]. Examining scholarly output with respect to the interactions of individual-level, building-level and department-level faculty attributes provides insights into the complex processes of knowledge creation. This is particularly salient in the contemporary research environment–one that is increasingly cross-disciplinary, collaborative, and reliant on digital communication tools [6,7].

We address these questions by focusing on the Massachusetts Institute of Technology (MIT), a community of scholars organized in various interrelated departments, labs, centers and institutes. Some of these bring researchers together in a dedicated building, some share a building between research groups, while others are institutional frameworks that link people who are separated by spatial distance or disciplinary boundaries. Considering several large datasets, comprising bibliometric and spatial information, we implement geospatial, network, and statistical analysis tools in a study of the MIT community over the ten-year period from 2004 to 2014. Our approach is a novel application of several emerging socio-spatial and network-based methods [4,8–13], applied to a research campus for the first time.

This paper investigates the following questions: How are physical and institutional organization on a university campus related to patterns of collaborative research among its faculty? Are these patterns comparable between papers and patents? Can we identify and characterize a relationship between physical distance of researchers and intensity of their collaboration, even at the micro-scale of a university campus?

To answer these questions, we apply empirical methods to a large dataset from MIT–a research institution that is exemplary in its collaborative scientific output. This paper arrives at an initial understanding of the fundamental relationships between physical location, institutional organization, and research outcomes.

The analysis reported herein reveals clearly diverging trends in the composition of collaborative teams between papers and patents, with relatively stronger institutional collaboration for papers and more pronounced cross-disciplinary collaboration for patents. We then construct a multi-layered network of authors [13], and find two significant features of collaboration on campus. Firstly, a network topology and community structure that reveals spatial versus institutional collaboration trends among co-inventors. Secondly and most importantly, we empirically demonstrate a persistent relationship between physical proximity and intensity of collaboration [14], well fit with an exponential decay model as already observed in collaboration networks at a larger geographic scale [11,15]. This relationship is consistently observable for both papers and patents, and is also consistent for exclusively extra-departmental collaborations. This is a particularly interesting result, in that it demonstrates the significant role that spatial proximity still plays in collaborative knowledge creation process–even at the micro (architectural) scale considered in this study, and despite the abundance of tools for digital communication and virtual collaboration. Our results point to the possibility of a natural ‘distance limit’ for collaboration, a hypothesis that could be validated by repeating the analysis across different campuses and research organizations.

Overall, our study paves the way for a set of further analyses to map complex evolving place-based networks. The analytical approach applied here–to study the interactions between space, collaboration, and academic performance–is of value as a replicable methodology, contributing a spatial dimension to the established field of scientometrics [6,16–23] and a network-science approach to space syntax and planning [24–26], bringing the two fields closer together. Furthermore, a multivariate analysis of scientific production and collaboration provides a better quantitative understanding of the MIT’s qualitative identity: both the pedagogical and architectural foundations of the institute emphasize cross-disciplinarity and a merger of theory and practice. Today, in the context of new buildings, research groups, initiatives, and labs with a stated ambition to support collaboration, it is crucial to build a body of empirical knowledge and spatial practices that reinforce the core innovation-driven ethos of MIT.

We propose that these analyses could, in the future, be operationalized–as a data-driven approach to institutional policy, architectural design and campus planning–with the potential to significantly impact on MIT. Furthermore, these frameworks may then be generalizable to a number of analogous contexts: academic campuses, research organizations, or neighborhood-scale business clusters [27,28]. We propose that a new approach to socio-spatial organization may ultimately enrich the design and operation of places for knowledge creation, supporting the cross-disciplinary collaborative activity that is increasingly vital to research and practice.

## Results

**Fig 1:**
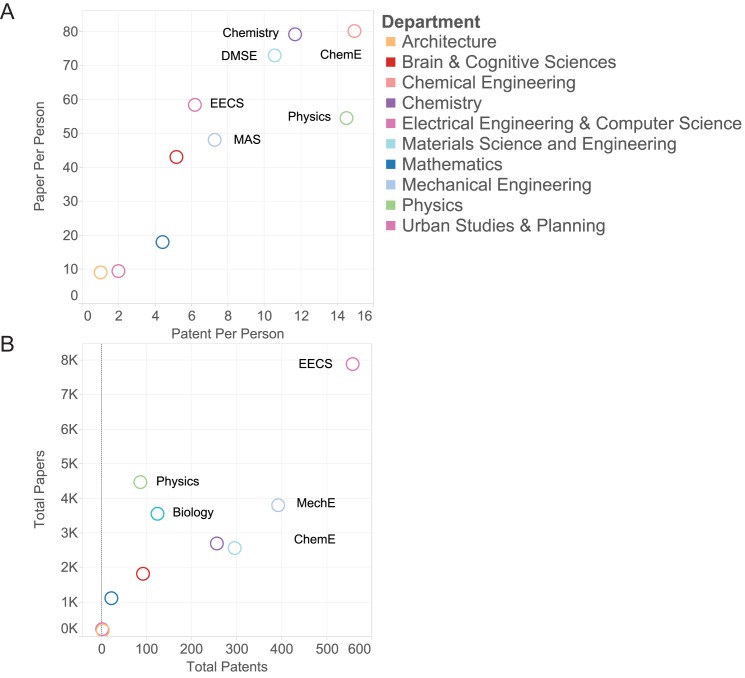
The ratio of paper vs patent output, aggregated to the department and individual levels. Each circle represents a department, and output per department / per person determines position along the axes. Many departments tend to have a bias toward one output type, but a notable group produces high relative numbers of both papers and patents. Here EECS, MechE and ChemE stand for Electrical Engineering and Computer Science, Mechanical Engineering and Chemical Engineering respectively.

We analyze the scholarly activity of the MIT community over the ten-year period from 2004 to 2014 considering more than 40 thousand scientific papers and more than two thousand patents (Fig 1).

This dataset, including MIT tenure and tenure-track faculty within the time frame, will be used for the remainder of the study. Table 1 summarizes the dataset, listing unique documents (papers and patents) as observations; the average annual output of the MIT community over the time frame; observations with more than one MIT faculty involved; total number of publishing and patenting faculty (not mutually exclusive).

**Table 1:** Summary of data after pre-processing. This dataset, including MIT tenure and tenure-track faculty between the years 2004 and 2014, will be used for the remainder of the study. The table lists unique documents as Observations; the average annual output of the MIT community over the time frame; observations with more than one MIT faculty involved; total number of publishing and patenting faculty (not mutually exclusive).

	Papers	Patents
**Observation**	40,358	2,350
**Avg Annual Number of Observation**	3,668.9	213.6
**Collaborative Observations with >1 MIT faculty member**	6,414	454
**Number of Faculty**	1,035	359

### Comparing paper and patent output

**Fig 2:**
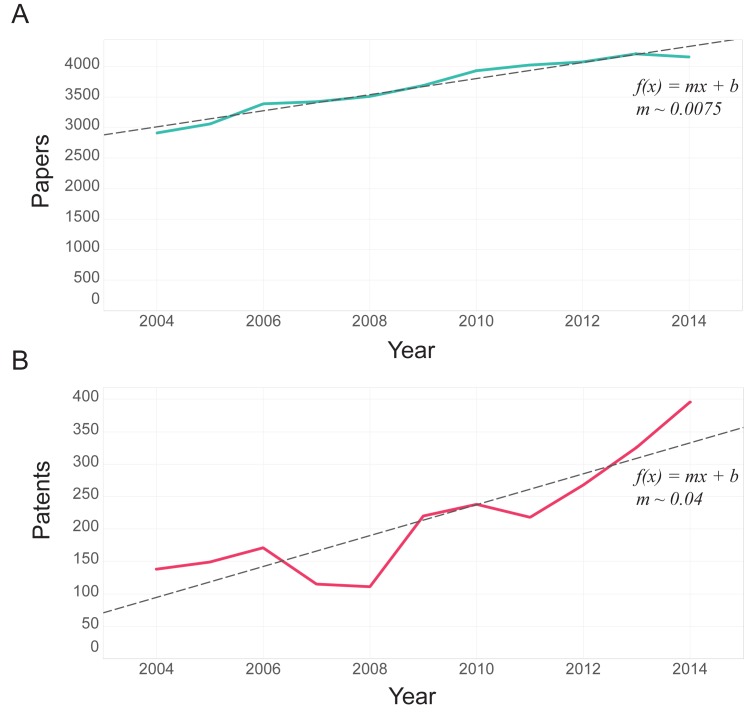
**Annual A) papers and B) patents output by MIT faculty, between 2004 and 2014, with trend lines describing growth.** A) Papers are fit with R^2^ = 0.96 p-value <0.0001 and t-value 14.12. B) A) Patents are fit with R^2^ = 0.77 p-value of 0.0004 and t-value of 5.45. The trends show that number of patents (a = 0.04) are growing faster than papers (a = 0.0075).

Between the years 2004 and 2014, overall scientific output from MIT increased. The rate of increase in publications remained relatively constant over time, while the rate of patent increase is more variable. Fit with a linear growth model (*R*^*2*^ = 0.96), paper output exhibits an average annual increase of 131.58 papers per year. Although it appears non-linear, we compare patents to papers assuming a linear growth model, and find an average annual increase of 23.84 patents per year (*R*^*2*^ = 0.77), both shown in Fig 2. These trends are statistically significant over the 99% confidence interval.

The MIT community has produced an increasing number of papers and patents per year, but absolute output numbers are very different between the two output types. We validate the difference in paper and patent activity and calculate the relative frequency of observations per year for both output types– considering total output of each as 1 and annual output for each year as a fraction of the total (Fig 2). On average, the patent output of the total MIT faculty community has been increasing at a relative rate of about 1% per year versus .3% per year for papers, respectively.

### Department level activity

In considering the entire MIT academic community (rather than a domain-specific subset) it is necessary to acknowledge variations between disciplines. Some fields produce knowledge that is inherently un-patentable, but those fields are no less active. A comparative analysis of scholarly output should account for– or at a minimum, distinguish between–fields and sub-fields of science (a common practice in scientometrics [9,20–23,29,30]). The results presented in this paper are not intended as a comparison between departments, but a summary of characteristic trends by discipline, which serves to inform the interpretation of subsequent results.

**Fig 3:**
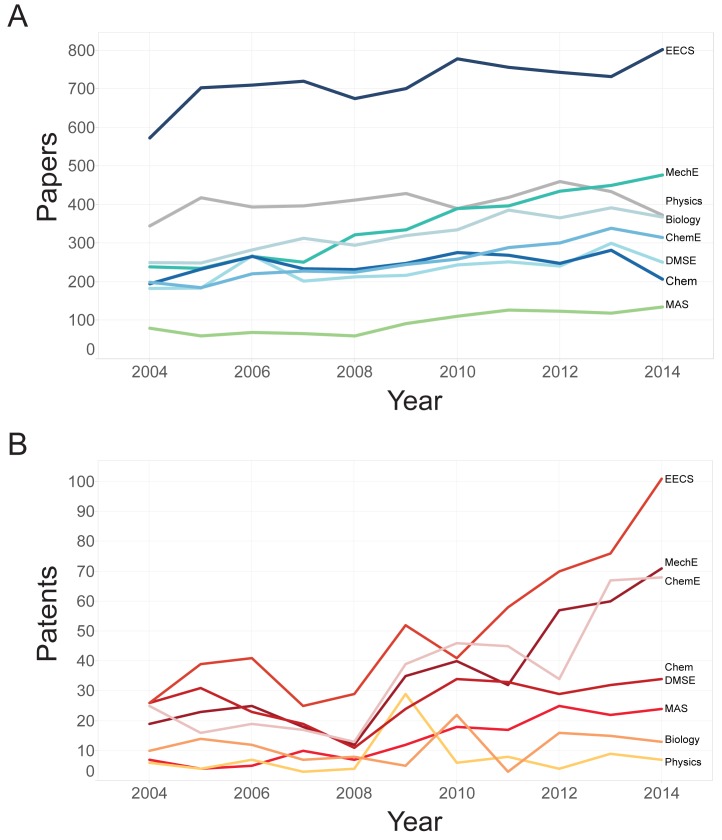
Patent and paper output per department per year. A) Paper output remains fairly constant at the department level, and as a trend across all departments. B) Conversely, some departments exhibit a strong increase in patenting while others do not–there is a notable increase in the disparity of output over the course of the decade (excepting a nearly universal dip in 2008). Here EECS, MechE, ChemE, Chem, DMSE and MAS stand for Electrical Engineering and Computer Science, Mechanical Engineering, Chemical Engineering, Chemistry, Dept of Material Science and Engineering and Mathematics respectively.

We characterize paper and patent output by 33 departments and research groups (an example of the highest producing departments, in aggregate, for each publication type is reported is in Table A in S1 File). The top producing departments follow very different patterns over time, for both papers and patents as shown in Fig 3.

The former maintains a fairly constant upward trend, within individual departments and across the entire publishing MIT community, while patenting is more variable. Some departments exhibit a strong increase while others do not, resulting in a divergence over time (excepting a nearly universal dip in 2008). The top five patenting departments have very similar output in 2004 (a range of less than ten patents per year), and vary dramatically over the following decade (to a range of over 80 patents per year). To better characterize the decrease in 2008 and verify that it is not an inconsistency in the data, we plot the same curve for the top patenting research institutions in the United States [41]– Stanford, CalTech and the UC system. A similar pattern appears, particularly in the UC system. Further investigation of the procedures and incentives for patenting suggests that this aberration may have been related to a bill passed by the United States Senate that changed the funding structure of the USPTO, causing a transition to a ‘second pair of eyes’ review system, and resulting in a sharp decrease in patent grants across the country. At the department level, there are meaningful trends in academic fields–for example, in 2012, over 50 MIT physicists (including 6 faculty members) were involved in the breakthrough discovery of the Higgs Boson particle [42], in collaboration with the European Organization for Nuclear Research (CERN). The discovery is widely regarded as one of the greatest scientific advances of the year, and resulted in a number of associated publications. It is also possible to observe, in a coarse way, the temporal disparity between paper and patent publication sequences– specifically, EECS exhibits a peak in paper output around 2010, and only in the years following (2011–2014) a steep growth in patent output.

### Mapping building-level data

**Fig 4:**
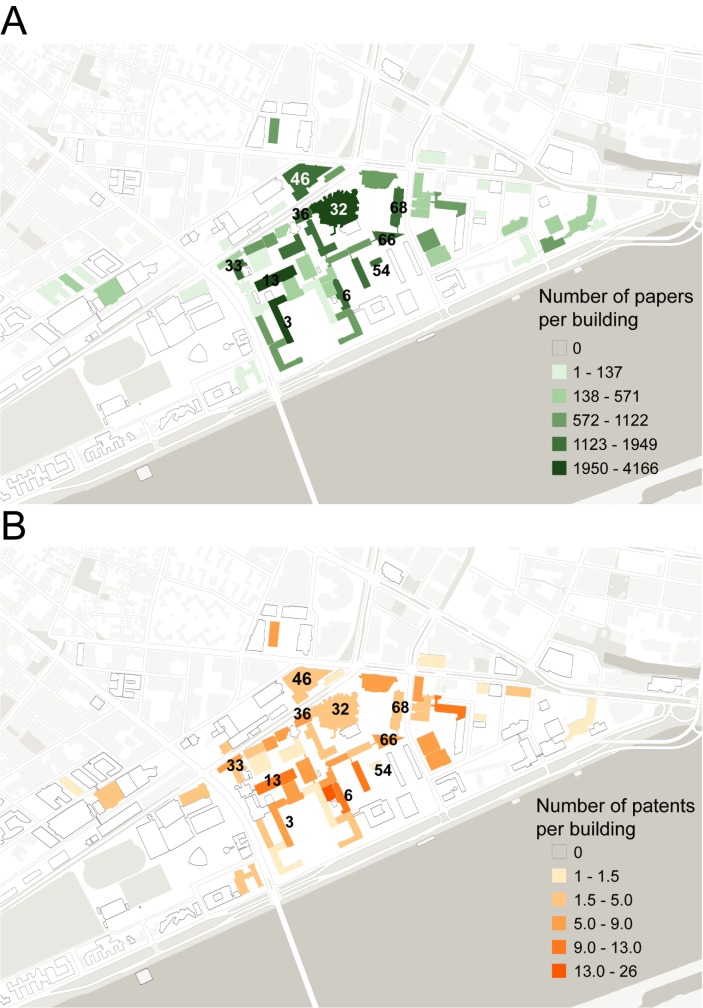
Paper and patent output per building between 2004 and 2014. On these choropleth maps, buildings are color coded by output volume and labeled with their name (the facility code). Colors are assigned using a Jencks algorithm with five buckets. (For a detailed list of the buildings see https://whereis.mit.edu).

Each building at MIT is uniquely allocated– some correspond one-to-one with a department, others are shared among several departments, and most departments are spread across several buildings. We begin by examining the distribution of scholarly output across the campus, aggregated to the building level. Fig 4 shows a choropleth map of campus with buildings coded according to aggregate volume of scholarly output for the decade.

Table C and Table D in S1 File show the top five buildings for each publication type, as well as the output per person and the collaboration ratio–a ratio of the number of documents with another MIT faculty member versus the number without. Maps of buildings coded according to output per person are shown in Fig 4. Considering annual output of buildings can evidence the effect of specific events (changes in funding, installation of equipment and research resources, or closures, for example) that may have influenced the rate and volume of publication. Fig A in S1 File shows building-level trends for paper and patent output from 2004 to 2014, counting only those observations that were created in a given building during a given year.

### Intra-building and intra-department collaboration

Many MIT buildings host a wide variety of departments, labs and research groups–more than sixteen distinct entities have a faculty member in Building 3, for example. We hypothesize that such spaces facilitate and promote collaborative activity, given that proximity and co-location are vital to research, particularly across disciplinary boundaries.

We first compare rates of collaboration between researchers who are co-located and those who are co-affiliated. To understand the composition of research teams, we define a metric of relative heterogeneity among the contributor group, based on the Simpson Diversity Index (see Section C in S1 File). Each observation (paper or patent) is assigned a value for building and department diversity among its contributors, using the Shannon entropy index (see S1 File for further details).

**Fig 5:**
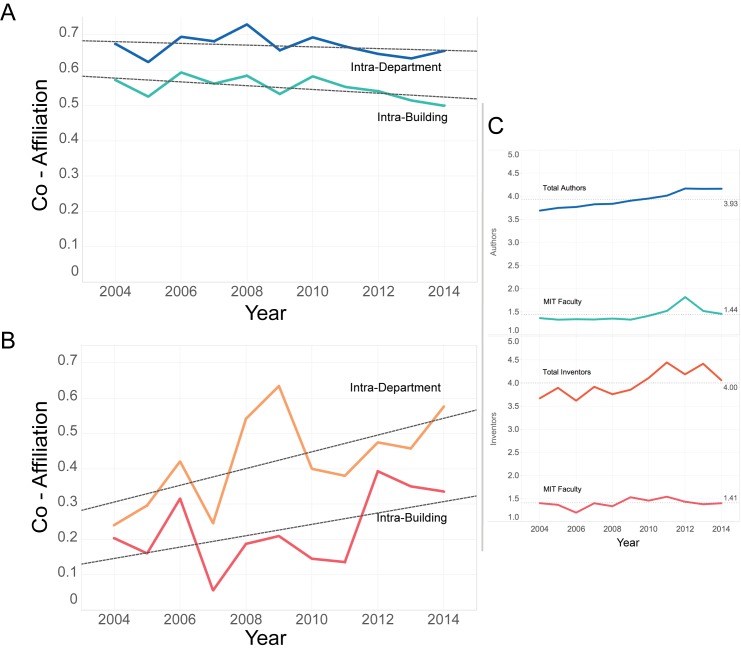
Intra-Building and Intra-Department activity. Each observation that has more than one MIT faculty collaborator is assigned a value (from 0 to 1) such that higher values indicate a greater incidence of co-affiliation, for either a building or department. Notably, rates of collaboration within buildings and departments have increased for patents, and decreased for papers. The latter is far more variable, and exhibited an intra-departmental peak in 2009, followed by an intra-building peak in 2012. A) For papers we observed an average of 0.55 for building and 0.67 for Departments. B) For patents we have an average of 0.23 and 0.42 for intra-building and intra-departments activity respectively.

Fig 5 shows the annual average co-affiliation index per observation, for those observations that are co-authored with another MIT faculty member (of a total 710 authors and 214 inventors collaborating internally within MIT). The rates of intra-building and intra-departmental collaboration on papers are roughly parallel over time. Patents, however, are more varied– intra-departmental collaboration spiked in 2009, and intra-building collaboration in 2012. As previously discussed, there was a universal dip in patenting activity around 2008. It is possible that, for those patents that were nonetheless granted in 2008–2009, there was an increased incentive to co-invent with faculty members from the same department.

It is important to contextualize the observed trends of intra-MIT collaboration reported in Fig 5A and 5B with respect to general collaboration trends (total authors, including those external to MIT in Fig 5C). Considering intra-MIT and total collaboration over the same time period, we observe that, for both papers and patents, the total number of collaborators on a given document shows a weak, positive trend between 2004 and 2014, while the number of MIT faculty has remained relatively constant (Fig 5C). Over the same time period, co-affiliation trends between MIT faculty collaborators is significantly different between papers and patents. The frequency of MIT co-authors sharing a departmental affiliation or building location has decreased over the time period (Fig 5A), while the frequency of co-inventors sharing a departmental affiliation or a building location has increased significantly (Fig 5B). Trends for co-invention are highly variable, differing between institutional affiliation and physical location, but support the increasing relevance of co-affiliation for patenting.

### Diverse spaces

**Fig 6:**
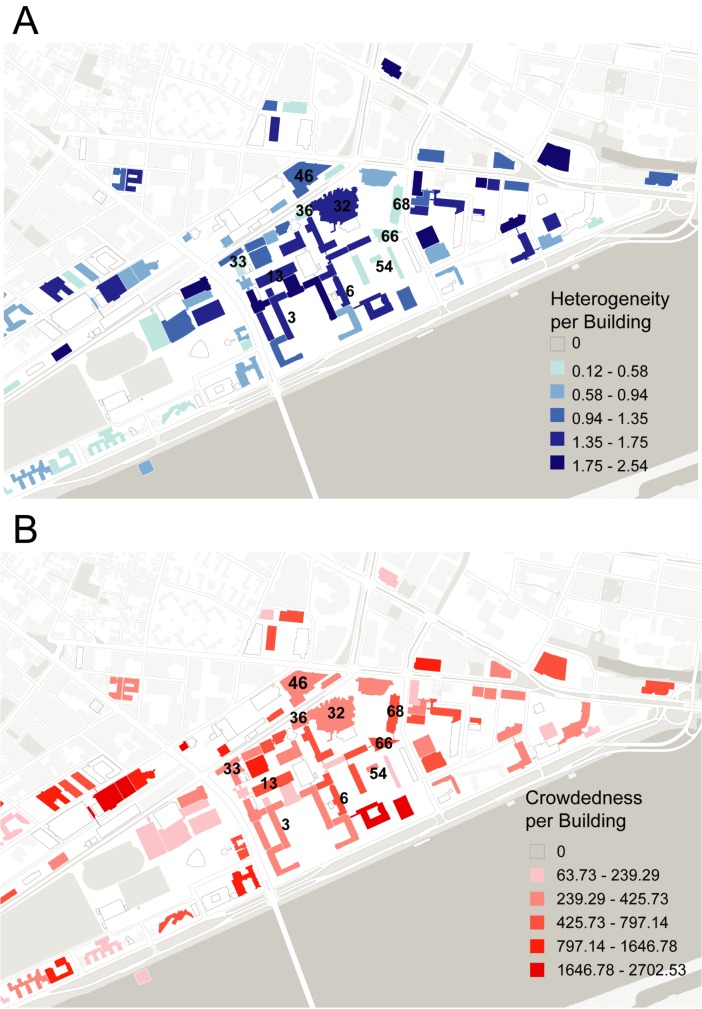
MIT Buildings Heterogeneity. A) Campus buildings, coded according to heterogeneity, as calculated using the Shannon measure of information entropy. This shows variation in faculty departmental affiliations per building. Values range from 0 to 2.5, classified with a Jencks algorithm. Buildings are labeled by name (facility code). B) MIT campus buildings, coded according the average total area of lab and office space per faculty member. There is a distribution of values from 145ft2 to 2,065ft2 allocated per faculty member. Buildings are labeled by name (facility code).

Not every building is occupied in the same way– some are allocated entirely to a single department or lab, while others host a diverse group of faculty from various disciplines. Buildings that have a higher degree of disciplinary heterogeneity may be areas of intellectual cross pollination. Heterogeneity of faculty affiliations can be quantified at the building level, defined as a function of the number of faculty and the number of different departments represented by those faculty members, using Shannon’s entropy of information (see Section D in S1 File for further details) [43]. As reported in Fig 6, we find a distribution of values between 0 (Building E62, which hosts 93 faculty members, all in 1 department), and 2.54 (Building 4, which hosts 8 faculty members in 4 departments) (Table C in S1 File). Using a correlation coefficient analysis, we examine whether there is a statistically significant relationship between building heterogeneity and academic output, but, contrary to the hypothesis, find none.

### A diverse crowd

Some buildings on MIT campus are both crowded and heterogeneous–spaces that could be considered intellectually vibrant. Those with the least space for labs and offices allocated to the most diverse group of faculty are shown in Table C in S1 File. However, there is no overall correlation with either paper or patent productivity–all are below the total averages of 6.50 and 0.85, respectively. Furthermore, there is no statistical correlation between diverse, crowded buildings and intra-building collaboration. Despite that there are no global correlations between heterogeneity and output or crowd and output at the campus level, there are nonetheless notable patterns in outliers for disciplinary diversity. The buildings with the largest absolute number of departments (for example, Building 3, with nine departments, or Building 32 with eight departments) are also outliers in academic output (this is true of total and per person output). Simply having a breadth of departments represented in the same building may promote collaboration, particularly across disciplinary boundaries. These buildings could be thought of as hubs on campus– a consideration that implies a campus ‘network effect.’

### The effect of institutional initiatives

While the analysis presented so far has revealed non-conclusive results concerning the effect of spatial organization on academic production, the data set allows quantifying the effect of various institutional initiatives at MIT that are aimed at fostering inter-disciplinary research in emerging fields.

Building 76, better known as the Koch Institute for Integrative Cancer Research, hosts such an interdisciplinary center for cancer research. The center was initiated through a $100 million grant from David H. Koch in October 2007, with a mission to bring together distinct approaches to cancer research. This first shift connected researchers who were formerly disparate in terms of affiliation, and provided funding for research partnerships. In December 2010, the new building was inaugurated, offering specialized equipment and facilities, and serving as a nexus for cross-disciplinary work.

Since its first patent in 2011, Building 76 quickly rose to be the top inventing building (Fig A in S1 File). Furthermore, the building has the highest rate of overall intra-MIT co-authorship, defined as the percentage of total publications that are with another MIT faculty member (Table A and Table B in S1 File). While restricted to a single example, our analysis hints to a clear success of institutional initiatives in bringing together researchers from different fields.

### Complex network analysis

We represent the MIT Faculty as a three-layer network *G* = (*N*, *L*^*1*^, *L*^*2*^, *L*^*3*^), where *N* nodes represent MIT Faculty Members and the *L*^*x*^, *x = 1*,*2*,*3* links represent one of three things: a collaboration (1. paper or 2. patent) between two nodes, or the 3. physical distance between them. (See Materials and Methods for detailed network generation process).

**Fig 7:**
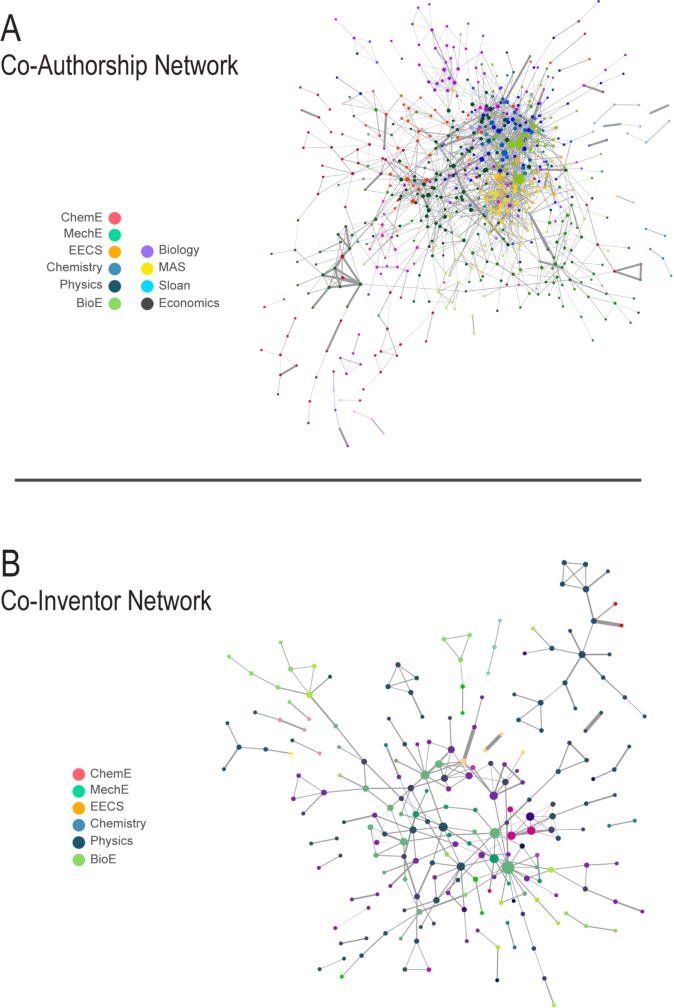
**A) Co-authorship and B) co-inventor networks.** Nodes represent faculty members, and node size is determined by its degree. A link indicates one or more collaborative papers or patents, and its thickness is the weight–a function of number of papers between the given authors, and the total number of authors on those papers. Nodes are colored according to department, and some notable departments are labeled. Here EECS, MechE, ChemE, BioE and MAS stand for Electrical Engineering and Computer Science, Mechanical Engineering, Chemical Engineering, Biological Engineering, and Mathematics respectively. Visualization has been done using Cytoscape.

An initial report of the size and characteristic properties of the co-authorship and co-invention networks is given in Table 2, including: node degree distribution, average clustering coefficient, and characteristic path length. Fig 7 is a visualization of the networks, with the following notable features: Average Number of Neighbors indicates that MIT authors who collaborate at least once with another MIT faculty member (and, as such, participate in the network) have an average of 5.47 MIT collaborators over the 10-year period, while collaborative inventors have an average of 2.51.

**Table 2:** Networks properties.

	Paper	Patent
**Number of Nodes**	710	215
**Transitivity**	0.233	0.234
**Connected Components**	19	22
**Diameter**	16	14
**Shortest Paths**	429820	19526
**Characteristic Path Length**	4.91	5.08
**Mean Degree**	5.47	2.51
**Average Betweenness Centrality**	0.016	0.131

The percentage of Shortest Paths, as well as the Average Betweenness Centrality, and the community structure of the two networks, are largely a result of the size and density of the networks. Finally, the Node Degree Distribution follows a power law for both networks, i.e. *P(x)~x*^*-γ*^, with *γ = 2*.*1* (see Fig B in S1 File), suggesting that they are scale-free, although the size of the patent network is too small to make strong claims about its scale-free properties[31]. Some conspicuous features are apparent in the visualizations– for example, the Department of Chemical Engineering tends to be very central to the network, with a high average degree, while Electrical Engineering & Computer Science is widely distributed, but with a low degree. Furthermore, some insular groups are evident, such as the Sloan School of Management and the Department of Economics. We find also find an average betweenness centrality of the co-invention network to be 0.131 and the co-authorship network to be 0.016 This suggests that for patenting, it is more common for certain faculty to be crucial links, as the shortest path between other patenting faculty. Members of communities in the paper network are more densely interconnected, while the patent network has key nodes with a high Betweenness Centrality.

### Community detection and network topology

The concept of ‘cross-pollination’ presumes distinct but interconnected groups. In network science [32,33], a cluster–or ‘community’–is defined as a group of nodes that have a higher probability of being connected to each other than to members of other groups (though other patterns are possible [34]). Our real-world network of collaboration at MIT is comprised of several such communities. Some are given–for example, faculty who are co-located in a building necessarily form a cluster in the spatial proximity network. However, communities can also be identified mathematically, as a property of the network. Effectively mapping the community structure of the networks reveals groups of faculty who work together, how those groups interrelate, and the ‘linking’ faculty that bridge disparate groups. The schematic cluster definition is not sufficient to identify structure– it is necessary to specify a threshold of community membership. Communities may overlap as well, sharing some of the nodes. For instance, in social networks individuals can belong to different circles at the same time, like family, friends, work colleagues [35–37]. We use the OSLOM algorithm– Order Statistics Local Optimization Method, an algorithm that accounts for edge weights, overlapping communities, hierarchies and community dynamics [38]–to detect the community structures of the networks (Fig D in S1 File).

Community detection in networks is a problem that is difficult to approach, as the definition of community itself is evasive and highly dependent on the context. Consequently, there are no clear-cut guidelines on how to assess the performance of different community detection algorithms.

We tested several community detection algorithms, and through a comparison of their performance, and decided to use Oslom. This algorithm is known to perform better with networks that have overlapping communities–as we expect to be the case for scientific collaboration [39], particularly within an academic institution.

The resulting network topology, algorithmically determined, can then be compared with the given building-level and department-level communities, to understand the relationship between collaboration and co-location or co-affiliation.

### The diversity of collaborative communities

Mapping a network that is clustered by communities of collaborative faculty is visually informative– for example, comparing a visualization in which nodes are colored by department with one in which nodes are colored by building. Moving beyond a general impression of diversity, we propose a simple quantitative measure of network topology based on assortment in community groups. The diversity of community structure in the network is given by the expression:

$$d_{i}=\frac{1}{N}\sum_{i}\frac{a_{i}}{p_{i}}$$ (1)

where *N* is the total number of communities, *p*_*i*_ is the number of members of community *i* and *a*_*i*_ is the number of affiliations in community *i*. A high value of *d*_*i*_ indicates greater diversity in the community structure of a particular network– showing, for example, that communities in the network tend to span across buildings more than they span across departments. We find that this is, in fact, the case. The co-authorship network is characterized by a *d* value of 0.40 for buildings and 0.29 for departments, and the co-invention network by *d* values of 0.51 and 0.70 (see Table D in S1 File). These attributes of network topology confirm the previous statistical results: building-diversity within collaborative communities is largely constant, but co-authors tend to favor collaborators in the same department, while inventors collaborate across disciplinary boundaries. Intuitively, this result points toward motivations for team composition– that co-inventors may collaborate around projects, while co-authors collaborate within domains of scholarship.

### Proximity and collaboration

**Fig 8:**
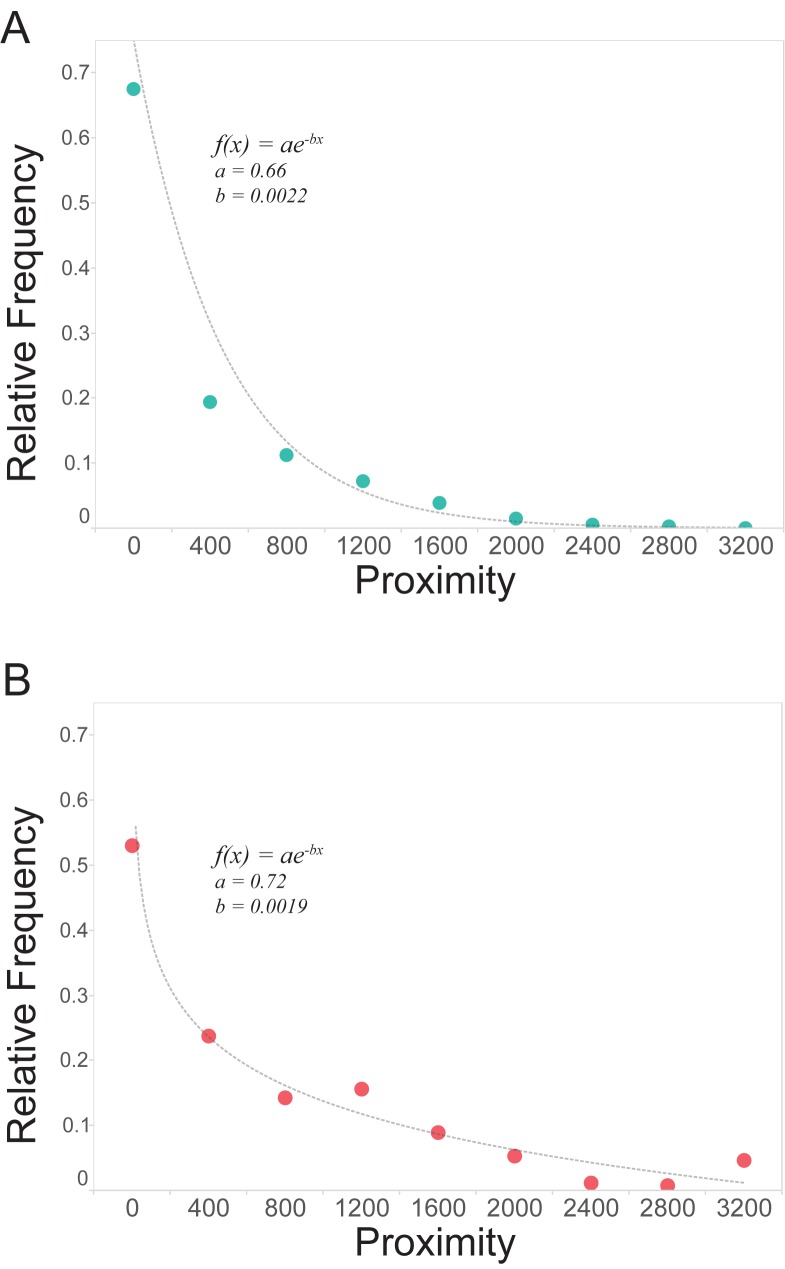
The relative frequency of collaborations between MIT faculty, plotted against their spatial distance on campus. A) Papers and B) Patents. As distance between two faculty members increases, the likelihood of their collaboration decreases according to a negative exponential function. The same pattern holds true for patents and papers. Papers are fit with R^2^ = 0.98 while patents with R^2^ = 0.99.

It is evident that there are strong patterns of collaboration related to departments and buildings–but does simple proximity have an effect? Using the physical distance between collaborators, we test the hypothesis that proximity is correlated with co-authorship and co-invention. Fully confirming this hypothesis, we find that the probability of a collaboration between two agents decays exponentially with their spatial proximity, as previously observed in research that addresses a larger spatial scale [11,15]. Probability adheres to a negative exponential function with a remarkable degree of consistency–not unlike, for example, the dissemination of dandelion seeds. One is more likely to find a seed close to the dandelion flower, and the likelihood of finding a seed decays exponentially as the distance increases. In the case of MIT faculty, one is more likely to find a collaborator close by. Co-authors have a higher tendency to collaborate at distance 0, suggesting co-authorship within a laboratory or department, while co-invention is more regularly spaced along the exponential curve (Fig 8). The probability *f*(*x*) is given by:

$$f(x)=ae^{-bx}$$ (2)

The parameters that fit with the highest correlation as shown in Fig 8.

### Proximity and cross-disciplinary collaboration

One could imagine that the relationship between proximity and output as evaluated above–and particularly co-authorship at distance value 0 –is a function of co-location in the same building. That is, that the relationship between proximity and collaboration may be skewed by tightly clustered and internally collaborative departments which are also co-located. To control for this, we plot the same proximity function, specifically for pairs of collaborators from different departments. As with the total dataset, the decay of collaboration frequency over space– even in the specific case of cross-departmental collaboration–is modeled by a negative exponential function. For both papers and patents, the fitting curve has a strong statistical significance (Fig 8). This clearly indicates that spatial proximity, even when not induced by institutional co-location, plays a definite role in academic collaboration.

## Discussion

Through statistical, geographic, and network analyses, we make a number of observations about socio-spatial collaboration dynamics within the MIT community. Between the years 2004 and 2014, MIT faculty produced over 40,000 papers and 2,300 patents, of which 6,414 and 454, respectively, involved more than one MIT faculty member. Our preliminary analysis confirmed underlying characteristics of academic disciplines: the fact of publishing a paper is different across fields, and some areas of academia simply do not produce patents–disparities that are clearly evident in a department-level analysis. In light of the literature on collaboration and co-location, we tested several building-level attributes– including central positioning on campus, crowdedness, and heterogeneity of department affiliations–and found that their effects are negligible. Our initial hypothesis was that centrally positioned, densely populated and multi-disciplinary spaces would be active hotspots of collaboration, but these building characteristics are not statistically correlated with productivity, using the measures applied in this study. This might be related to the fact that the outcome of a multi-disciplinary collaboration is not necessarily a larger academic output. Multidisciplinary research outlets are often more selective than disciplinary ones, suggesting that the impact of multi-disciplinary collaboration should be measured considering a broader range of metrics. Contrary to the literature and intuition, building-specific factors (such as heterogeneity, crowdedness, etc.) had no significant effect on scientific output of MIT faculty, suggesting that our strategy for quantifying those factors may be incomplete. Nevertheless, we observed that those buildings with the greatest absolute number of departments are also outliers in productivity and collaboration. These outliers, then, can be understood as hotspots, despite that there is no global linear correlation. Over the 10-year period of analysis, the MIT community as a whole published and patented with an increasing number of total collaborators on each document. The rate of collaboration between MIT faculty has remained relatively constant, save for three notable exceptions: in 2009 and 2014 there was more co-invention among MIT faculty, and in 2012 there was more internal co-authorship.

A topology-based evaluation of collaborative networks reveals the composition and interaction of collaborative clusters. In the publication network, communities are more strongly related to departments, whereas in the patent network, communities are more heterogeneous overall, and have a tendency to align with buildings. This suggests that co-inventors organize around ideas or projects, while co-authors organize around disciplinary areas– a finding that reconfirms the statistical results related to team composition. Overall, these results reflect faculty members’ practical motivations for working together: co-inventors collaborate around projects, benefitted by shared equipment and a breadth of expertise, while co-authors collaborate within domains of scholarship to advance the knowledge of a particular subject.

Finally, we tested the effect of spatial proximity on collaboration. Regardless of co-location in a specific building, we found a persistent relationship between spatial proximity and the frequency of collaboration, well fit with an exponential decay model. Building on that observation, we suggest the possibility of a significant ‘distance limit’ for co-authors and an optimal ‘goldilocks distance’ between inventor pairs.

Moving forward, the present analysis can be supplemented in a number of ways. Alternate analytical designs, such as a difference in difference [4,40] approach, could more effectively isolate variables. Furthermore, introducing time in the network analysis (a dynamic system approach) can better show community formation, and exploring possibilities for evaluating the processes of collaboration, in addition to its products (for example, by creating another network layer from digital communication among faculty members) can address collaboration not observed in papers or patents. Together, these detailed analyses can provide an understanding of academic dynamics over time and space, revealing the impact of spatial and institutional configurations on collaborative research between faculty members at MIT. Socio-spatial network analysis is of practical interest to MIT’s scientific community, to campus planners and administrators, and to the individual faculty members who are engaged in research activity. Empirical analysis of interrelated networks and academic output on this campus advances the broader knowledge of complex relationships between architecture, institutions and collaboration. These results suggest opportunities for future research. We propose that the methods implemented for the present analysis could be applied to larger datasets that span across several research institutions or shifts in spatial and physical organization, to arrive at a comprehensive and causal answer to the research questions. The methods and results are particularly salient in the context of increasingly team-based science and emphasis on cross-disciplinary research.

## Materials and methods

We posit that the spatial organization of MIT faculty is an important factor of the knowledge creation process, and examine the ways in which this is manifest in scholarly output, including rate, volume, authorship and collaboration patterns of publishing and patenting. To approach this question, we investigate a number of sub-hypotheses:

1. Building heterogeneity (co-location of faculty from different departments) is positively correlated with cross-disciplinary collaboration, both internal and external to the building.
2. Faculty tend to collaborate within buildings, and with colleagues who have a higher degree of spatial proximity (a negative correlation between distance and probability of collaboration) even if collaborators are in different departments.
3. The community structure of the co-authorship network has a higher degree of similarity to departments or initiatives, and the community structure of the co-invention network has a higher degree of similarity to buildings.

Anecdotally, we experience certain buildings as collaborative hubs– remarkably active, densely and heterogeneously populated spaces. These play an important role in initiating contact among researchers, specifically those from different disciplines who would not otherwise meet. As such, we suggest that researchers sited in hub spaces will exhibit a higher instance of cross-disciplinary collaboration.

Similarly, because spatial proximity–regardless of specific building location–is an important determinant of regular communication and a crucial element of successful collaborative work, we propose that it will be associated with higher incidence of collaboration, regardless of the specific building. These characteristic socio-spatial patterns will be different between papers and patents, as a result of the inherent disparity between collaboration processes (related to the use of technical equipment, research lab structure, and differences the definition of publishable novelty), disciplinary fields, and academic norms for attribution between papers and patents. Acknowledging that difference, we emphasize that the purpose of this study is not to demonstrate the differences between scientific output types.

Three original databases are implemented in the present study: 1. Institute Directory; 2. Publication; 3. Patent. These three databases are linked using the MIT Identification Number: a unique 9-digit numerical value assigned to each MIT affiliate, which persists through changes in affiliation over time. The linked data is limited, by time and scope, to ensure precision, as detailed below. The truncated data is then processed to derive second-order variables (e.g. a faculty member’s ratio of papers to patents), as well as aggregated (e.g. to the department or building level) and linked to auxiliary data (e.g. mapped to building footprints to calculate geodetic distance between faculty). The final dataset spans 2004–2014 and encompasses 40,358 papers, 2,350 patents, and 1,057 MIT faculty. Datasets and key attributes are summarized in Table 1. An extensive description of the database used in this research and the data processing analysis is reported in Section B in S1 File.

### Complex network analysis

#### Layer 1: Proximity

As distinct from ‘centrality,’ we define ‘proximity’ as an attribute of faculty collaborator pairs *f*_*ij*_. Using floor (*F*) and building (*B*) attributes of each faculty member, a proximity value *p* can be calculated for every link in the MIT faculty collaboration network. The proximity index is a hierarchical distance function that accounts for both distance between buildings *ΔB*_*ij*_ (measured in linear feet between building footprint centroids), and difference in floors *ΔF*_*ij*_

Let *i* and *j* be two nodes (faculty) in the MIT collaboration network, and define *ΔB*_*ij*_ as the geodesic distance between the centroids of buildings *B*_*i*_ and *B*_*j*_ where *i* and *j* are located. Furthermore, let *ΔF*_*ij*_ = | *F*_*i*_-*F*_*j*_ | be the floor difference between the location of *i* and *j*, where *F*_*x*_ is an integer representing the floor in building *B*_*x*_ where *x* is located. We introduce a scale factor, *d*, to account for the difference in units of measure between *ΔF*_*ij*_ and *ΔB*_*ij*_ and calculate *d* such that:

$$d({\max\left(\Delta F\right)}_{\left\{mn,\ldots .,ij\right\}})<{\min\left(\Delta B\right)}_{\left\{mn,\ldots .,ij\right\}}$$ (3)

The proximity value *p*_*ij*_ is then defined as:

$$p_{ij}=\begin{array}{c} d(\Delta F_{ij})\;if\;B_{i}=B_{j}\\ \Delta B_{ij}+d(F_{i}+F_{j})\;otherwise \end{array}$$ (4)

For each edge *f*_*ij*_ the proximity index *p*_*ij*_ is a measure of difference in floors *ΔF*_*ij*_ and geodetic distance between centroids of buildings *ΔB*_*ij*_. Acknowledging that linear horizontal distance between buildings is experientially very different from vertical difference between floors, we nonetheless define the proximity value as a function of the two. A scale factor of *d* is introduced to adjust floor values such that they are comparable to distance values. However, for the scope of the present study, we do not attempt an empirical space syntax to better synthesize multiple distance metrics [25,26]. Defining a better quantitative measure of functional distance is a promising avenue of further research.

#### Layer 2: Co-authorship index

The publication value is summarized as a scalar index of scientific productivity. Each node corresponds to one author *i* and a link between two authors *i* and *j* exists if and only if the two authors have collaborated at least one time on the same paper. Links are weighted with a value representing the intensity of collaboration between the respective actors. Given that the purpose of the network analysis is to better understand spatial relationships–for example, co-location and frequency of contact between collaborators–we include the total number of co-authors, presuming that the spatial dynamics of collaboration between a team of two is different from the dynamics between a team of ten [20].

The publication edge weight (*b*_*ij*_) accounts for the number of co-authored papers between faculty *i* and *j* and the number of total co-authors for those papers (MIT faculty and non-MIT faculty). As such, the weight of a link summarizes not only co-productivity between collaborators, but also the way in which those collaborations were carried out. Suppose that two authors *i* and *j* have collaborated *n*_*ij*_ times in *n*_*ij*_ different papers, then the weight *w*_*ij*_ is given by:

$$w_{ij}=\frac{n_{ij}}{\sum_{k=1}^{n_{ij}}\left(n_{k}^{c_{ij}}-1\right)}$$ (5)

Where $n_{k}^{c_{ij}}$ is equal to the number of total co-authors on paper *k*.

#### Layer 3: Co-invention index

Similar to co-publication, a weighted link is used to represent intensity of co-patenting activity. A summary of the size and characteristic properties of the co-authorship and co-invention networks is shown below, and network degree distribution is reported in Fig B in S1 File, while detailed networks features are reported in Table 2 including: node degree distribution, transitivity, and characteristic path length. Notable features include the following: Average Number of Neighbors indicates that MIT authors who collaborate at least once with another MIT faculty member (and, as such, participate in the network) have an average of 5.47 MIT collaborators over the 10 year period, while collaborative inventors have an average of 2.51. Furthermore, the percentage of Shortest Paths, as well as the Average Betweenness Centrality, evidence differences in the community structure of the two networks. Finally, the Node Degree Distribution follows a power law for both networks (Fig B in S1 File), suggesting that they are scale-free, although the size of the patent network is too small to make claims about its scale-free properties [31]. Some conspicuous features are apparent in the visualizations below– for example, that the Department of Chemical Engineering tends to be very central to the paper network, with a high average degree, while Electrical Engineering & Computer Science is widely distributed, but with a low degree. Furthermore, some groups emerge, such as the Sloan School of Management and the Department of Economics.

## Supporting information

S1 File Supplementary text. This text contains an extensive literature review, a complete description of the data analysis procedure, the formula of the Simpson Diversity Index and the Shannon Entropy of Information. Supplementary Tables and Figures are included at the end.
(PDF)
