# Supplementary material for: An exploration of collaborative scientific production at MIT through spatial organization and institutional affiliation
Source: PLoS One. 2017 Jun 22;12(6):e0179334. doi: 10.1371/journal.pone.0179334 (PMC5480888; doi:10.1371/journal.pone.0179334)
Supplement: S1 File — This text contains an extensive literature review, a complete description of the data analysis procedure, the formula of the Simpson Diversity Index and the Shannon Entropy of Information. Supplementary Tables and Figures are included at the end. (PDF) [file pone.0179334.s001.pdf]

Supporting Information

An Exploration of Collaborative Scientific Production at MIT Through Spatial Organization and Institutional Affiliation

Authors:

Matthew Claudel[1,2 ¶*], Emanuele Massaro[2¶*], Paolo Santi[2,3], Fiona Murray[4] and Carlo Ratti[2]

Affiliations:

[1]Lab for Innovation Science & Policy, Massachusetts Institute of Technology, Cambridge, MA 02139

[2]Senseable City Laboratory, Massachusetts Institute of Technology, Cambridge, MA 02139

[3]Istituto di Informatica e Telematica del Consiglio Nazionale delle Ricerche, 56124 Pisa, Italy.

[4]MIT Sloan School of Management, Cambridge, MA 02142

*Correspondence to: claudel@mit.edu, emassaro@mit.edu

[¶]These authors contributed equally to this work.

Supplementary Information

Section A – Extensive Literature Review

Scales of Collaborative Activity, from the Desk to the Globe

A. Microgeographies: The Desk

The configuration of personal workspaces – at the scale of a desk in a room – certainly define social interactions. Those configurations may also define the types of ideas that are collaboratively generated, through increasing exploration during low opportunity-cost time, and enabling accelerated iteration during early or uncertain phases of collaboration [1,2]. Yet in the case of workplace microgeography, it is often difficult to identify cause and effect: whether researchers work nearby because they collaborate, or if they collaborate because they work nearby. To address concerns of endogeneity in microgeographic organization, studies have been designed to exploit a random exogenous factor – for example, the unexpected spatial reconfiguration of researchers. Over the course of a move to new premises, Peponis [3] conducts survey-based social and spatial network analysis, finding that layout can contribute to frequency and volume of communication. A similar analytical design by Catalini (focusing on reconfigurations during building closure for asbestos screening), demonstrates that, beyond simply provoking communication, shifts in proximity lead to increased experimentation and eventually to stronger 'breakthrough ideas' [1].

B. Hubs: The Building

The majority of the literature reviewed previously – that addressing lab configurations, office proximity, space syntax, collaboration and re-configuration – falls within the architectural scale.

C. Ecosystems: The District

Although it has no discrete boundary or categorical definition, the 'district' is perhaps the most relevant scale for innovation processes with respect to commercialization. There is a rich literature from urban planning, management, organization science and economics, as well as an emerging field of innovation science, that considers this so-called 'ecosystem' or 'cluster' – an area smaller than a city, and more heterogeneous than a campus. Topical literatures have emerged around specific terms and definitions, but all address this district scale. These include 'Marshallian industrial districts,' 'innovative milieus,' 'new industrial spaces,' 'innovation networks,' 'regional network evolution,' and most recently, the 'ecosystem-approach' [4,5]. Each has a unique classification – for example the 'cluster' definition is a geographic concentration of interconnected companies and associated institutions in a particular field, linked by commonalities and complementarities [4,6,7].

This work – and the management science literature in general – primarily addresses market-related characteristics and effects of proximity. Porter's seminal work on entrepreneurial clusters, for example, describes inter-firm relationships, and evaluates such phenomena as the competitive transfer of financial and human capital. In contrast, the present analysis focuses on interrelations, knowledge-creation and technology transfer outside of (or prior to) commercialization, but nonetheless accesses the literature describing general effects of geographical and institutional proximities (campus space and departments, respectively).

The innovation ecosystem, as defined in the MIT Stakeholder Model is a vibrant, co-located agglomeration of high-growth potential firms and related stakeholders, an interconnected set of people, resources and the physical environment that provides the context for innovation-driven enterprises to start, grow and scale. The model describes place-based interrelations of five key stakeholders: entrepreneurs; universities or research institutes; government; corporations; and risk capital.

D. Agglomerations: The City

Understanding collaboration and innovation at the urban scale (and the inter-urban, national and global scales) demands fundamentally different tools and procedures. It is at the metropolitan level that data becomes big data, beyond this threshold it can only be approached through a specialized set of statistical,

social, mathematical and computational analysis techniques – from the collection, management and processing of data, to the application of statistical models, to means for addressing concerns of subject privacy, and even to visual representation and dissemination.

Intuitively, metropolitan-scale conclusions can be drawn using this approach. Stern and Guzman, for example, interact a number of publicly available data points related to new firms (including name, keywords, intellectual property, legal status, etc.) and construct an ex-ante model of entrepreneurial quality: the statistical likelihood of success outcomes, defined as acquisition or IPO. The resulting Regional Entrepreneurship Cohort Potential Index (RECPI) can be trained on metropolitan regions to estimate the impact of a city itself on its firms: the Regional Entrepreneurship Acceleration Index (REAI) measures the performance over time as the ratio between the statistical success potential of a cohort of firms and their observed success rate in that city. This tool has been used to evaluate and compare cities across the United States, finding characteristic patterns of entrepreneurship [8].

Even more generally, certain urban effects related to collaboration and knowledge output are quantifiable through a big data analysis approach. For example, a number of metropolitan phenomena – including patent filings, for example – scale superlinearly with city size [9]. An empirical model proposed by researchers at the Santa Fe Institute is consistent across cities, time periods and nations. Derivative growth equations evidence a number of urban taxonomies, for example, the distinction between urban growth fueled by entrepreneurial innovation versus growth through economies of scale, as measured by wealth and patents [10]. Trends in collaborative invention are significantly spatialized as well. Large metropolitan areas tend to have a higher number of patents and larger team sizes: an average of greater than three co-inventors. The most highly productive metropolitan areas have over four inventors per patent [11]. In the case of inter-urban and global collaborations, a characteristic distance – unique to specific fields – can be quantified [12].

These, and a number of related studies, access scientometric observables (for example, collaboration, publication density, co-inventor distance, and others) and apply macro-level tools of urban science, delivering valuable insights at a low level of spatial granularity. For the purposes of the present research, city-scale dynamics are important for contextualization, but specific results are not easily transferrable to the campus or building scale.

Observing Scholarship: Faculty, Papers and Patents

Proximity-enabled communication – whether in the lab, the building, the campus, the institution, or the cluster – is a crucial enabler of scientific knowledge creation. Place is at the core of the university model throughout history. Yet for intellectual material to be recognized, validated, and built upon or commercialized, it must be codified and disseminated to the global scientific community, specifically, as papers and patents: observable knowledge objects. Although these documents are not comprehensively representative of intellectual activity at a given institution (and their role varies dramatically across disciplines), they nonetheless offer a systematic proxy for scholarly output. Bibliometric data enables empirical analysis of impact, affiliation, collaboration, and citation over time.

For the purposes of this study, we consider papers published in peer-reviewed journals, and patents filed through the MIT Technology Licensing Office, but a portrait of scholarly activity at MIT should be contextualized by broader trends in scientific production. In the case of paper publishing by the global scientific community, individual productivity (on both single-author and collaborative observations) has a fat-tailed distribution: a small number of scientists produce a very large number of papers, and this has been consistent over time (from Lotka's Law of Scientific Productivity, proposed in 1926, to Newman's network analysis in 2001 [13]). Synthesizing several estimations of bibliometric data, Borner proposed a general model for the growth of scientific publishing as well as co-authorship and citation trends, successfully validated against twenty years of publication data from the Proceedings of the National Academy of Sciences [14]. The global rate of increase in scholarly publication (estimated as the number of cited papers that are subsequently cited) is approximately 8% [15].

The World Intellectual Property Organization (WIPO) estimates a similar growth for intellectual property. Excepting a downturn during the global economic crisis in the years 2008 and 2009, the rate of increase in patenting is now approximately 9.2%. College, university, and institute patents represent between 4.2 – 4.7% of U.S. nongovernmental patents, of which Massachusetts Institute of Technology holds an annual average of 4.2% (of all U.S. patents granted to U.S. academic institutions).

The Porous Walls of the Institution: Managing Technology Transfer

A categorization of research is particularly relevant with respect to incentives and procedures for publication, implementation and commercialization. Technology transfer is an important economic driver, and foregrounds the role of intellectual property rights policies [16]. The pathway from research to commercialization was fundamentally redefined in 1980 with the Bayh-Dole Act (or Patent and Trademark Amendments Act) that is in place today. The act systematized and unified patent policy across federal agencies, and allowed for universities and research institutes to hold patents that result from government funded research. This legal measure shifted incentives for researchers of all levels to patent and license through the institution, and and for the organization itself to engage in joint research ventures or enable spinoff startup companies. An increase in the commercialization rate of university-based technology following 1980 can be attributed in some part to the Bayh-Dole Act.

A more explicit approach to technology licensing has given rise to the 'economics of intellectual property' at universities and research institutions [17]. Many institutions now have technology transfer offices that serve to manage the exit dynamics of scientific research. Although terms are unique to each organization, in most cases there are clear motivations for faculty to disclose and file patents through the institution, at various stages of technical resolution, from proof of concept to product [18].

At MIT, the Technology Licensing Office (TLO) assists the passage of research from the lab to the market, through patenting and licensing. The TLO interfaces with large corporations, SMEs and startups ¬– brokering the legal use of patented MIT research – and generally assists students, faculty and staff with their pursuit of innovation-driven work. The TLO files patents with USPTO, and has worked with an average of 20-27 new firms per year from 2000 to 2014.

Time Delays in Academic Output

There is a significant time delay in the course academic production from idea to publication. The sequences for papers and patents are categorically different, resulting in a significant discrepancy between overall time delay (a discrepancy generative of certain experimental structures, e.g. Murray and Stern, 2007 [19]). Although there is variation across fields, papers are accepted, on average, 6.4 months after submission, and are published 5.8 months after acceptance – the total average time delay is 12.2 months from submission to publication in a journal. The patenting process is substantively different, and tends to vary widely between different contexts. In the case of MIT, an individual approaches the TLO with a patent application, and works with the office to revise the document. A formal application is filed by TLO at the US Patent and Trademark Office between 0 and 5 months later, depending on revisions. There is then a period of 24-36 months before review by a patent examiner, followed by a granting decision period of 12-24 months. The total time elapse from submission to granting is an average of 2.5-5 years.

Section B - Data Analysis

Original Data: Directory

Directory information is from the MIT Directory (accessed in the MIT Data Warehouse, via the Office of Institutional Research). The full dataset includes all MIT affiliates, including students, researchers, fellows, tenure track and non-tenure track faculty, etc. The MIT directory contains location-attributes for offices (building, floor and room), as well as affiliation attributes (school, department, or lab), but the information is not recorded for every person (see B2). The MIT Data Warehouse commits a regular 'freeze' of the data, such that time-dependent information is captured (e.g. changes in affiliation or title).

Original Data: Publication

Publication information is from a comprehensive list aggregated by Academic Analytics, a non-institutionally affiliated data analytics company. Academic Analytics aggregates publication data from scholarly journals, for the purposes of evaluation, strategic decision-making, and benchmarking in universities. Publications in the Academic Analytics database have been consistently filtered with a proprietary algorithm to match individual authors, and the company's total database includes over 270,000 individuals from over 10,000 departments in 385 universities.

MIT-specific data has been acquired by the Office of Institutional Research (OIR) and is stored in the MIT Data Warehouse. The dataset includes papers published by MIT-affiliated individuals in peer-reviewed journals with DOI number identifiers, as well as date, and authorship. Data is stored such that a paper is associated with an MIT ID, and the same paper is repeated if there are more than one MIT authors on the paper. There are 65,536 MIT authorship instances (with repeated DOIs) and 52,511 papers (with unique DOIs) spanning the years 1959 to 2015. In this dataset, there are 1,440 total MIT authors (faculty and non-faculty).

Original Data: Patent

Patent information is from the database of patent applications filed through the MIT Technology Licensing Office (TLO) and aggregated in the MIT Data Warehouse (via the OIR). The dataset includes a unique identifier (Patent Serial Number) for each patent, as well as date of filing, MIT ID of primary inventor, and total authorship (all inventors listed on the patent). There are 7,344 patents, and 5,487 MIT inventors (faculty and non-faculty) over the years 1938 to 2015.

Pre-Processing Data

Through pre-processing the total databases, several necessary limitations and filters have been introduced to increase accuracy of the data under consideration. These filters are: B1. Time Frame (2004 – 2014); B2. Affiliation (Tenure and Tenure-track Faculty) to arrive at a final dataset, C.

Pre-Processing: Time Frame

The temporal limitation has two reasons. First, there was a sharp increase in the number of recorded papers in the year 2003, which may have resulted from Academic Analytics' migration to a digital database for content management. Although there are a small number of papers recorded as early as 1959, trends and attributes are not consistent before and after the shift. Secondly, the practice of regular data 'freezes' committed by OIR / MIT Data Warehouse began in 2004. In the interest of understanding changes over time (e.g. hiring or departing, affiliation, promotion, office location) rather than working under the assumption that a faculty member's current status is true of past years, we consider only this range, limiting the data to the time frame 2004-2014.

Pre-Processing: Affiliation

There are numerous modes of affiliation to MIT, many of them involved with the processes of research and the production of academic output. For example, an undergraduate student may participate in groundbreaking research and be listed as an author on a paper with her Principal Investigator. A comprehensive portrait of scholarly activity at MIT would capture these myriad interactions.

However, several attributes are consistent only for a subset of the MIT community – the result both of structural factors and data management. The former is intuitive: in the case of the UROP, she would not have a designated office location or even a departmental affiliation. The same is true, to varying levels of consistency, up to the level of post-doctoral fellows, lecturers, or professors of the practice. Furthermore, the data 'freeze' only captures attributes for certain levels of affiliation. The subset with full consistency

is tenure and tenure track faculty, who are necessarily affiliated to a department and located in an office. As such, the most accurate and consistent directory includes only faculty who are tenure or tenure track.

Data Summary

The dataset under consideration for the remainder of the study is characterized in Table 1.

Spatial Data

Spatial data describing the MIT campus is joined from several different sources. Building shapefiles (in GIS format) are from the Department of MIT Facilities and overlaid on publicly available map tiles (from OpenStreetMaps via CartoDB). Building-level data (area, facility use codes, office numbers and floors) are from the MIT Data Warehouse. The databases are linked using building code (e.g. Bulding E70; Building 9).

Section C - Simpson Diversity Index

For a given number of types, the value of a co-affiliation index (C) is maximized when all types are equally abundant. In this case, the number of species i = 1, 2, ..., d are defined by the total number of unique departments or buildings, and the number of different unique collaborators ni who belong to a given type i. If an observation is characterized by N authors and d types, the diversity index is given by:

$$C = \frac{1}{N(N-1)}\sum_{i=1}^{d} n_i(n_i - 1) \qquad \text{(Eq. S1)}$$

Where the co-affiliation value (C) of a single observation is a function of total number of contributors (N), from a total number of departments (d), and (ni) number of contributors from each of those departments (i). Values are on a scale from 0 to 1, representing least to greatest co-affiliation or co-location among collaborators.

Section D - Shannon Entropy of Information

The measure was originally proposed to quantify entropy in strings of text: the greater the difference in letters, and the more equal their proportional abundances in the string of interest, the more difficult it is to correctly predict which letter will be the next one in the string. The Shannon equation is applicable more broadly to quantify balance of information content, accounting for variety and quantity. In the case of MIT campus, we apply the following function to characterize buildings. Given a building *k* its heterogeneity index λ_k is then defined as:

$$\lambda_k = -\sum_{i=1}^{D_k} p_i \ln p_i \qquad \text{(Eq. S2)}$$

Where D_k = number of different departments in building *k*, and p_i is the fraction of people who belong to the department *i.* It is defined as:

$$p_i = \frac{N_{ik}}{N_k} \qquad \text{(Eq. S3)}$$

Where N_{ik} is the number of people that belong to department *i* in the building *k,* while N_k are the total number of people working in building *k*.

Section E – Tables

Department	Patent to Paper Ratio	Number of Faculty Authors	Total Papers / Person	Total Papers	Average Annual Increase	Intra-MIT Collab.	Number of Faculty Inventors	Total Patents / Person	Total Patents	Average Annual Increase	Intra-MIT Collab.
**Biology**	0.036	60	59.28	3557	14.52	20.4%	33	3.79	125	0.34	21.6%
**Chemical Engineering**	0.132	35	80.17	2806	14.47	25.3%	26	14.96	389	5.03	34.2%
**Chemistry**	0.096	34	79.12	2690	2.87	15.3%	22	11.68	257	1.7	30.0%
**Electrical Engineering & Computer Science**	0.069	135	58.47	7893	13.95	25.2%	90	6.2	558	6.25	30.1%
**Materials Science and Engineering**	0.117	35	73.00	2555	7.76	25.1%	28	10.57	296	1.03	30.7%
**Mechanical Engineering**	0.098	79	48.06	3797	26.51	17.8%	54	7.26	392	5.09	18.9%
**Physics**	0.019	82	54.52	4471	3.85	29.8%	6	14.5	87	0.25	78.2%
**Program in Media Arts & Sciences**	0.135	22	47.41	1043	7.72	12.7%	17	8.88	151	2.2	5.3%

Table A. Patent and paper output by department, for the top six departments in each category. *Patent to Paper Ratio* represents the total number of patents from a department divided by the total number of papers, such that 0 represents a department without patents and 1 a department without papers. *Intra-MIT Collaboration* is calculated as an average of the number of documents with another MIT faculty member vs. the total number of documents, such that values closer to zero represent a lower rate of intra-MIT collaboration. *Number of Faculty* enumerates the total number of publishing faculty from a given department, during the time frame. *Average Annual Increase* represents the slope of the trend line in output per year, a measure of the average increase in output year-to-year, by department

Building	Papers per Building	Intra-MIT Collaboration	Papers per Person	Patents per Building	Intra-MIT Collaboration	Patents per Person
**32**	4166	27.8%	110.0	140	30.7%	3.68
**3**	2570	19.3%	93.0	290	21.4%	8.29
**13**	2370	27.5%	78.9	278	45.7%	12.64
**36**	1949	19.6%	78.4	171	19.3%	7.77
**6**	1704	20.5%	77.4	157	42.0%	13.08
**66**	1662	28.8%	71.8	169	46.2%	8.45
**46**	1607	13.6%	67.2	69	7.2%	3.63
**54**	1372	12.2%	60.5	1	100%	1
**68**	1330	24.1%	59.4	22	9.1%	2.44
**33**	1285	16.8%	59.2	40	25.0%	4.44

Table B. Patent and paper output per building, for the top ten buildings, during the entire time frame. *Output per Person* represents the average individual output aggregated to the building level. *Intra-MIT Collaboration* is calculated as the number of documents with another MIT faculty member versus the total number of documents (aggregated to the building level), such that values closer to zero represent a lower rate of intra-MIT collaboration.

				Papers			Patents		
**Building**	**Depts.**	**Shannon Entropy**	**Average ft.^{2}**	**% MIT Collab.**	**Papers Per Person**	**Papers**	**% MIT Collab.**	**Patents Per Person**	**Patents**
**3**	9	1.56	340.50	19.3	47.59	2570	21.4	8.29	290
**E40**	8	2.06	256.80	17.4	13.08	340	-	-	-
**32**	8	1.71	398.53	27.8	39.68	4166	30.7	3.68	140
**16**	8	1.44	175.80	26.6	31.92	830	23.9	6.77	88
**E25**	7	1.64	479.15	16.8	36.33	981	17.5	10.36	114
**7**	6	2.45	350.54	12.5	4.80	48	-	-	-
**1**	6	1.28	344.08	12.3	29.85	1015	35.7	1.56	14
**76**	6	0.89	358.75	31.7	28.52	713	27.2	7.52	158
**E38**	5	2.28	354.48	21.1	6.33	57	100	1.00	1
**10**	5	1.99	145.95	19.9	18.17	527	44.6	6.22	56

Table C. MIT campus building attributes. Although there are no global correlations between heterogeneity and the observed indicators of academic output, there are nonetheless specific correlations between number of departments and output (notably, Buildings 3 and 32). Shown here are the ten buildings with the highest number of departments. *% MIT Collaboration* represents the percentage of observations that are with an MIT faculty collaborator versus the total number from that building.

**Diversity**	**Papers**	**Patents**
**Building**	0.40	0.51
**Department**	0.29	0.70

Table D. Community diversity is a measure of the number of different buildings or departments represented in each group, normalized by community size.

Section F – Figures

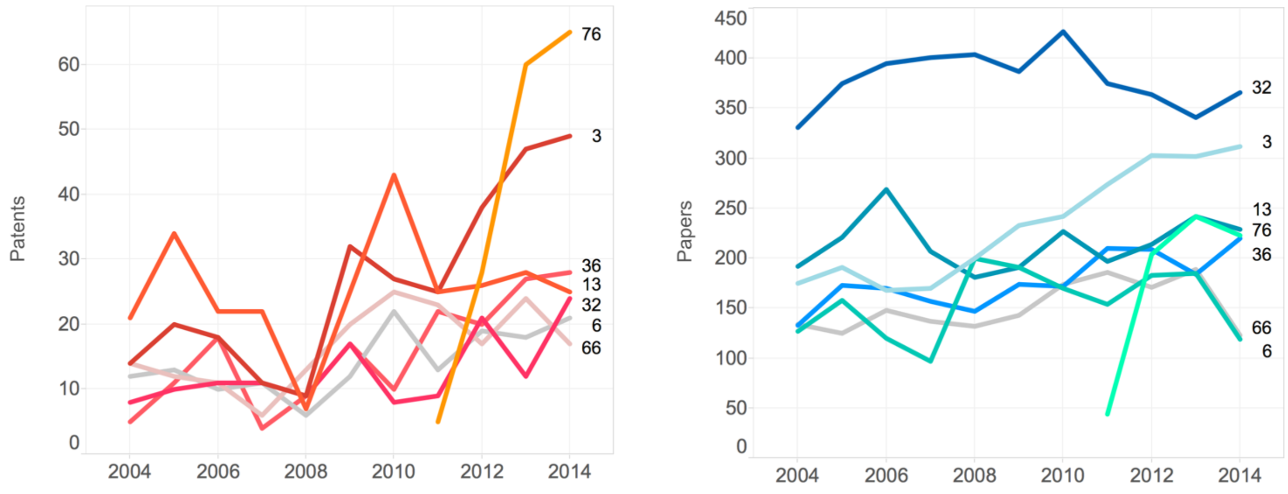

Figure A. Patent and paper output per building per year, from 2004 to 2014. This represents output from faculty during the year they were sited in a particular building – accounting for spatial relocations. Building 76, the Koch Institute, was opened in 2010 and by 2013 had become the top patenting building.

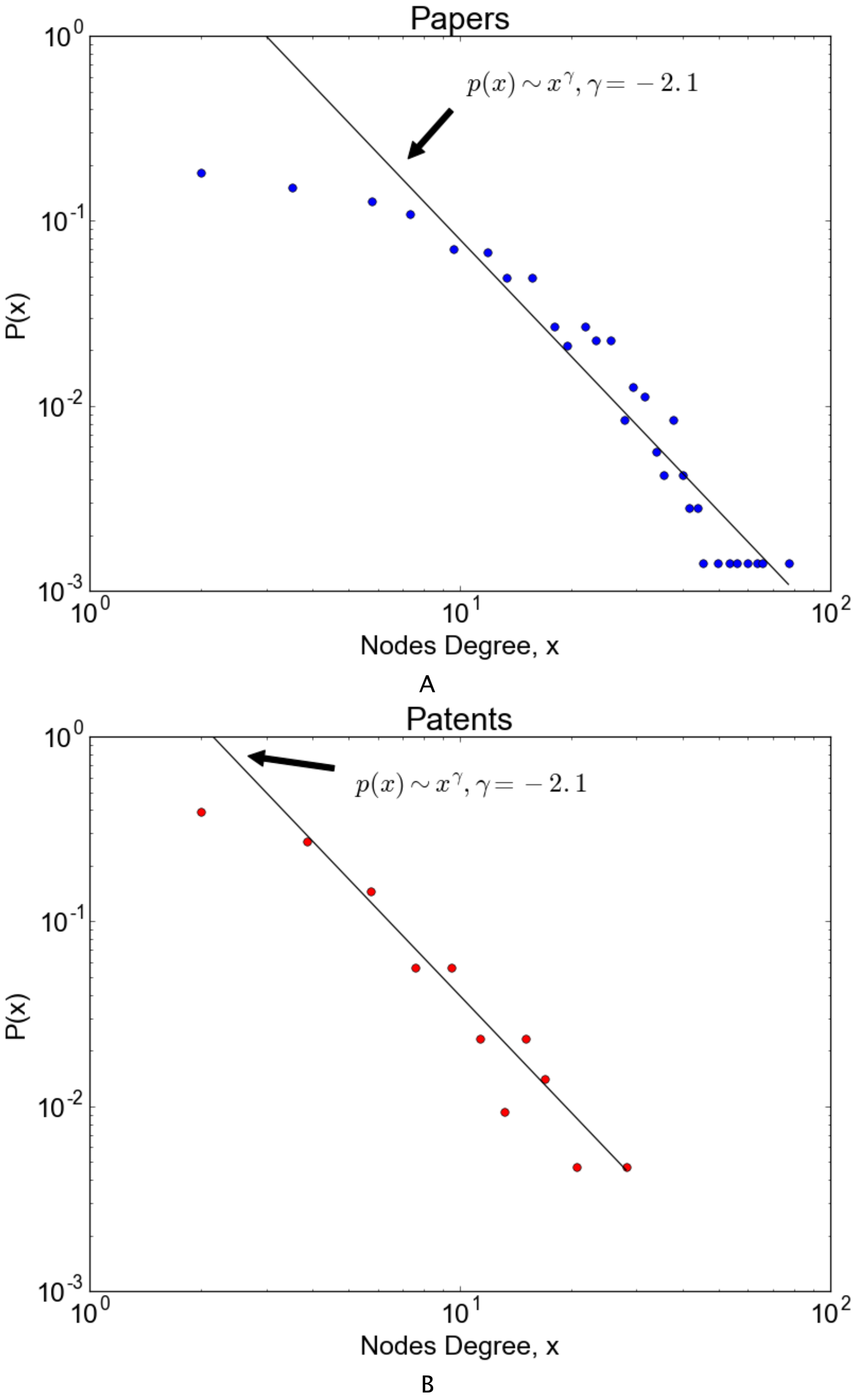

Figure B. Nodes degree distributions for A) Paper s and B) Patents networks.

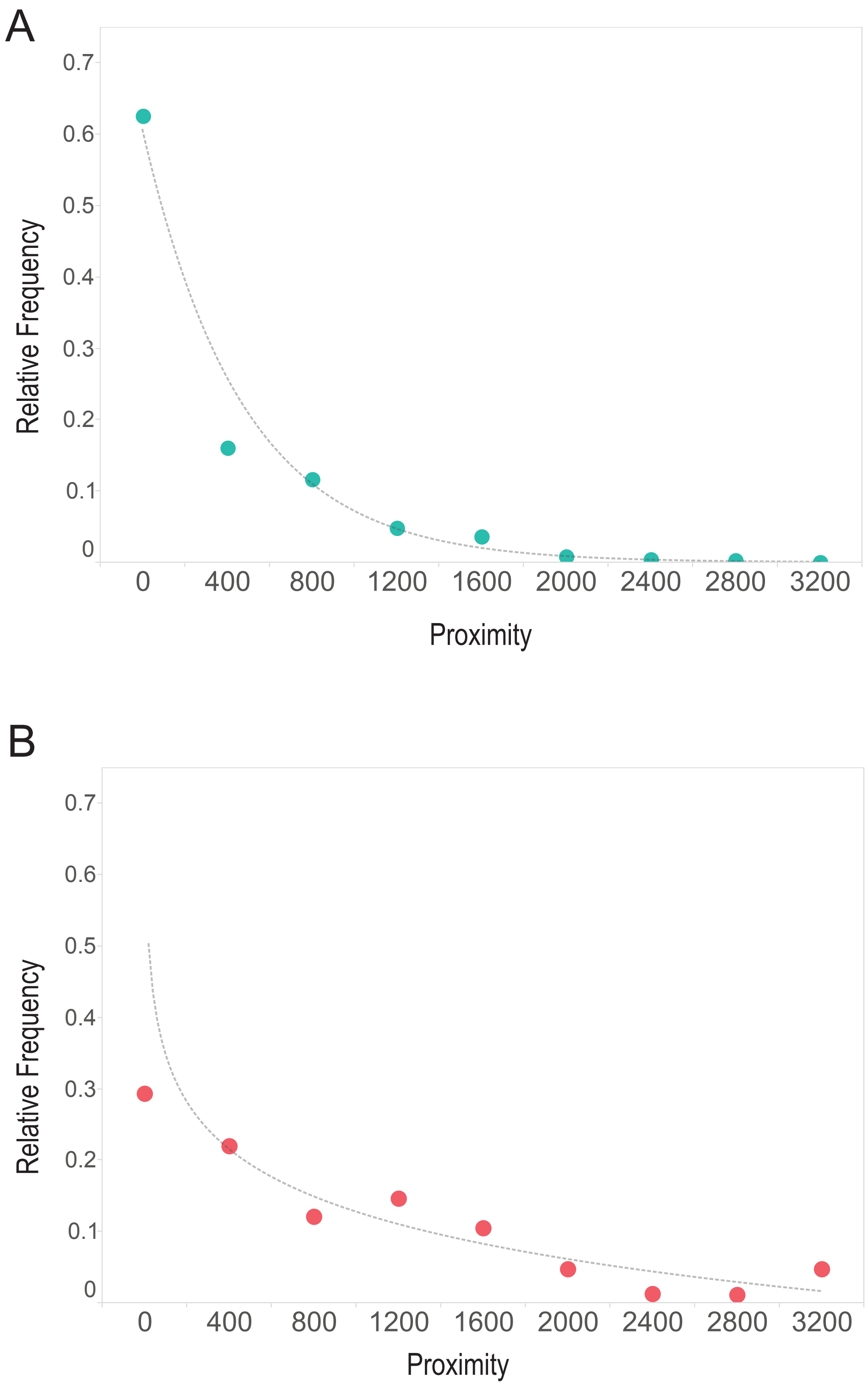

Figure C. The relative frequency of collaborations between MIT faculty from different departments, plotted against their spatial distance on campus. A) Papers and B) Patents**.** As distance between two faculty members increases, the likelihood of their collaboration decreases according to a negative exponential function. The same pattern holds true for patents and papers.

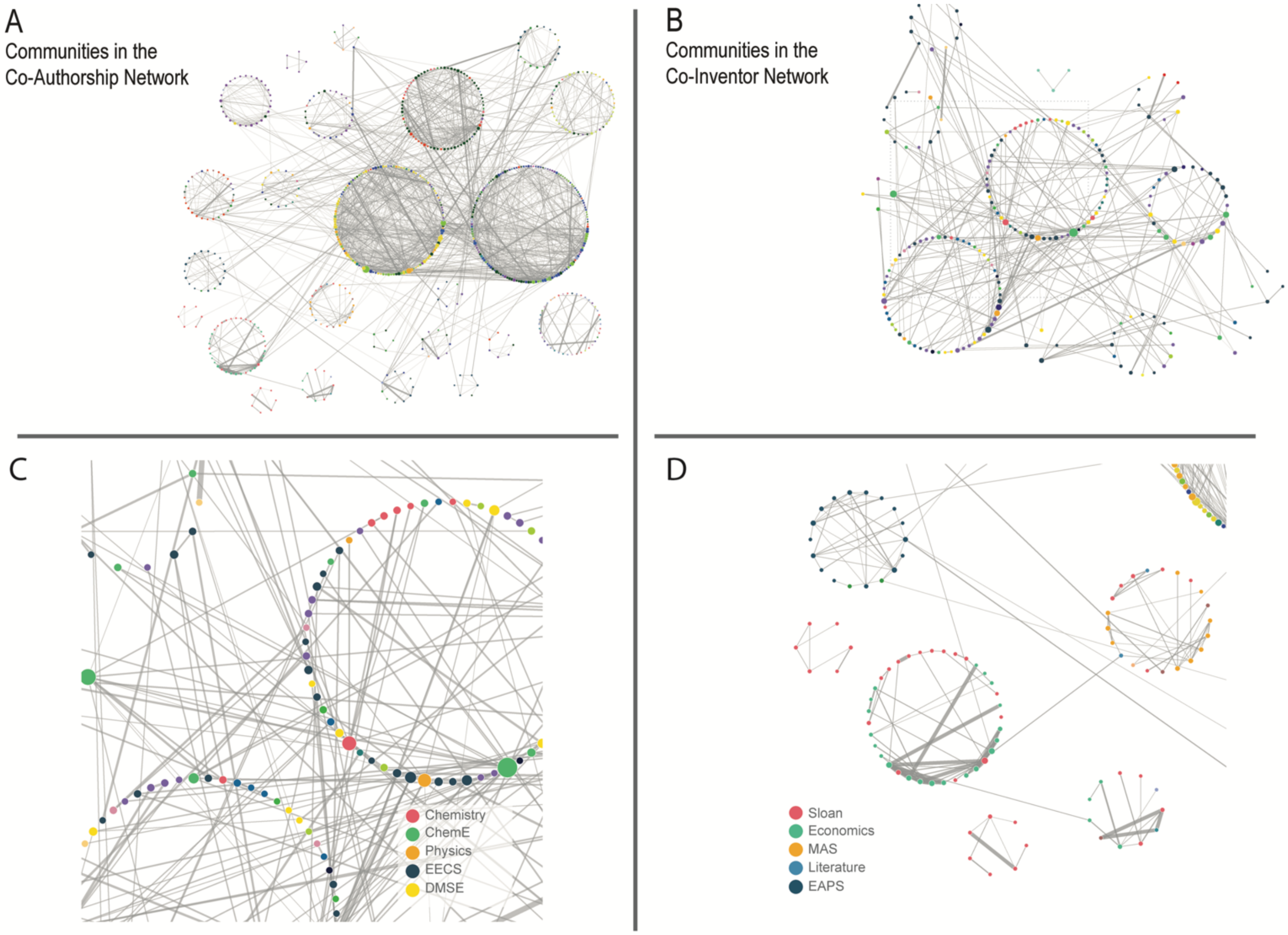

Figure D. Communities in the A co-authorship and B co-invention networks. C) The detailed views of the communities in the co-authorship network shows that seem to be more topically defined, in this case, Earth Atmospheric & Planetary Sciences, or Sloan and Economics**.** D) Communities in the co-invention network are more heterogeneous, comprising faculty from several different departments.

Section G - Bibliography

1. Catalini C. Microgeography and the Direction of Inventive Activity. SSRN Electron J. 2012; doi:10.2139/ssrn.2126890

2. Miron-Spektor E, Gino F, Argote L. Paradoxical frames and creative sparks: Enhancing individual creativity through conflict and integration. Organ Behav Hum Decis Process. 2011;116: 229–240. doi:10.1016/j.obhdp.2011.03.006

3. Peponis J, Bafna S, Bajaj R, Bromberg J, Congdon C, Rashid M, et al. Designing Space to Support Knowledge Work. Environ Behav. 2007;39: 815–840. doi:10.1177/0013916506297216

4. Porter ME. The competitive advantage of nations: with a new introduction. New York: Free Press; 1998.

5. Porter M, Stern S. Innovation: Location Matters. MIT Sloan Manag Rev. 2001;42: 28–36.

6. Glaeser EL, Kerr WR, Ponzetto GAM. Clusters of entrepreneurship. J Urban Econ. 2010;67: 150–168. doi:10.1016/j.jue.2009.09.008

7. Delgado M, Porter M, Stern S. Clusters, Convergence, and Economic Performance [Internet]. Cambridge, MA: National Bureau of Economic Research; 2012 Jul. Report No.: w18250. Available: http://www.nber.org/papers/w18250.pdf

8. Guzman J, Stern S. Where is Silicon Valley? Science. 2015;347: 606–609. doi:10.1126/science.aaa0201

9. Bettencourt LMA, Lobo J, Helbing D, Kuhnert C, West GB. Growth, innovation, scaling, and the pace of life in cities. Proc Natl Acad Sci. 2007;104: 7301–7306. doi:10.1073/pnas.0610172104

10. Bettencourt LMA, Lobo J, Strumsky D, West GB. Urban Scaling and Its Deviations: Revealing the Structure of Wealth, Innovation and Crime across Cities. Añel JA, editor. PLoS ONE. 2010;5: e13541. doi:10.1371/journal.pone.0013541

11. Rothwell J, Lobo J, Strumsky D, Muro M. Patenting Prosperity: Invention and Economic Performance in the United States and its Metropolitan Areas. Brookings Institute; 2013 Feb.

12. Tijssen RJW, Waltman L, van Eck NJ. Collaborations span 1,553 kilometres. Nature. 2011;473: 154–154. doi:10.1038/473154a

13. Newman MEJ. The structure of scientific collaboration networks. Proc Natl Acad Sci. 2001;98: 404–409. doi:10.1073/pnas.98.2.404

14. Borner K, Maru JT, Goldstone RL. The simultaneous evolution of author and paper networks. Proc Natl Acad Sci. 2004;101: 5266–5273. doi:10.1073/pnas.0307625100

15. Bornmann L, Mutz R. Growth rates of modern science: A bibliometric analysis based on the number of publications and cited references. J Assoc Inf Sci Technol. 2014;

16. Rosenberg N, Nelson RR. American universities and technical advance in industry. Res Policy. 1994;23: 323–348. doi:10.1016/0048-7333(94)90042-6

17. Link AN, Scott JT, Siegel DS. The economics of intellectual property at universities: an overview of the special issue. Int J Ind Organ. 2003;21: 1217–1225. doi:10.1016/S0167-7187(03)00080-8

18. Jensen R, Thursby J, Thursby M. The Disclosure and Licensing of University Inventions [Internet]. Cambridge, MA: National Bureau of Economic Research; 2003 May. Report No.: w9734. Available: http://www.nber.org/papers/w9734.pdf

19. Murray F, Stern S. Do formal intellectual property rights hinder the free flow of scientific knowledge? J Econ Behav Organ. 2007;63: 648–687. doi:10.1016/j.jebo.2006.05.017
